# Lipid production in batch and fed-batch cultures of *Rhodosporidium toruloides* from 5 and 6 carbon carbohydrates

**DOI:** 10.1186/1472-6750-12-26

**Published:** 2012-05-30

**Authors:** Marilyn G Wiebe, Kari Koivuranta, Merja Penttilä, Laura Ruohonen

**Affiliations:** 1 VTT Technical Research Centre of Finland, P. O. Box 1000, Espoo, FI-02044 VTT, Finland

**Keywords:** Lipid, *Rhodosporidium toruloides*, Bio- and renewable diesel, Fed-batch, High cell density

## Abstract

**Background:**

Microbial lipids are a potential source of bio- or renewable diesel and the red yeast *Rhodosporidium toruloides* is interesting not only because it can accumulate over 50% of its dry biomass as lipid, but also because it utilises both five and six carbon carbohydrates, which are present in plant biomass hydrolysates.

**Methods:**

*R. toruloides* was grown in batch and fed-batch cultures in 0.5 L bioreactors at pH 4 in chemically defined, nitrogen restricted (C/N 40 to 100) media containing glucose, xylose, arabinose, or all three carbohydrates as carbon source. Lipid was extracted from the biomass using chloroform-methanol, measured gravimetrically and analysed by GC.

**Results:**

Lipid production was most efficient with glucose (up to 25 g lipid L^−1^, 48 to 75% lipid in the biomass, at up to 0.21 g lipid L^−1^ h^−1^) as the sole carbon source, but high lipid concentrations were also produced from xylose (36 to 45% lipid in biomass). Lipid production was low (15–19% lipid in biomass) with arabinose as sole carbon source and was lower than expected (30% lipid in biomass) when glucose, xylose and arabinose were provided simultaneously. The presence of arabinose and/or xylose in the medium increased the proportion of palmitic and linoleic acid and reduced the proportion of oleic acid in the fatty acids, compared to glucose-grown cells.

High cell densities were obtained in both batch (37 g L^−1^, with 49% lipid in the biomass) and fed-batch (35 to 47 g L^−1^, with 50 to 75% lipid in the biomass) cultures. The highest proportion of lipid in the biomass was observed in cultures given nitrogen during the batch phase but none with the feed. However, carbohydrate consumption was incomplete when the feed did not contain nitrogen and the highest total lipid and best substrate consumption were observed in cultures which received a constant low nitrogen supply.

**Conclusions:**

Lipid production in *R. toruloides* was lower from arabinose and mixed carbohydrates than from glucose or xylose. Although high biomass and lipid production were achieved in both batch and fed-batch cultures with glucose as carbon source, for lipid production from mixtures of carbohydrates fed-batch cultivation was preferable. Constant feeding was better than intermittent feeding. The feeding strategy did not affect the relative proportion of different fatty acids in the lipid, but the presence of C5 sugars did.

## Background

An increased use of bio- and renewable diesel as replacement for petroleum-derived diesel in recent years has led to increased interest in the production of microbial lipids for fuel use; the aim being to produce lipids in micro-organisms using substrates which do not directly compete with food uses, as plant oils do. Both phototrophic and heterotrophic micro-organisms have been considered. Among heterotrophic micro-organisms, the optimal production organism would be able to use the carbohydrates present in plant biomass, cellulose and hemicellulose, or the C5 and C6 sugars released by hydrolysis of plant biomass, primarily glucose and xylose, but also including arabinose, galactose and mannose.

Numerous yeast and filamentous fungi accumulate high concentrations of lipids in their biomass and yeast belonging to the species *Rhodosporidium* (or *Rhodotorula*), in particular *R. toruloides*, have often been used as model organisms for lipid production. Concentrations of lipid over 50% DW are generally reported [1]. Both glucose and xylose are metabolised [2]. Most experiments have included yeast extract and/or peptone in the medium [2-6]. Although these are a convenient source of complex nutrients for rapid growth at lab scale, their cost may be prohibitive for a bulk product on an industrial scale and ammonium sulphate or the cheaper corn steep solids/liquid (CSS/CSL, respectively) could be preferable alternatives. Biomass hydrolysates will themselves supply trace elements, but their complex nitrogen may not be readily available.

Glucose is the most commonly supplied carbohydrate in basic studies of lipid production in yeast [1]. Sucrose, xylose, fructose, galactose and maltose [2,5,7] have also been studied, but there is only one report of lipid production from arabinose [1], even though it contributes 1 to 18% of the total carbohydrates in plant hydrolysates. Production of lipids from substrate mixtures have only been reported for xylose with glycerol [8,9], glycerol with glucose or xylose, or xylose with glucose [6,10]. Waste waters [11-13] and hydrolysates [5,14] are increasingly being used directly for lipid production.

An industrial lipid production process should make use of high cell density cultures, but the oxygen demand of the process limits the biomass density obtainable in small scale, flask cultures. High cell density cultures have been achieved for *R. toruloides* and *Cryptococcus curvatus* using fed-batch and repeated batch techniques [4,15,16]. Various feeding strategies have been described. Hassan et al. [15] demonstrated that 70 g L^−1^ biomass containing 53% (w/w) lipid could be achieved with *C. curvatus*, at ~0.2 g L^−1^ h^−1^, in a culture started with C/N ~10 but fed a solution of glucose not containing nitrogen. Li et al. [4] similarly used a batch culture with a low C/N, fed with nitrogen free glucose solution to obtain high biomass (106 g L^−1^) and lipid production (0.5 g L^−1^ h^−1^) of *R. toruloides* Y4 in complex medium. Discontinuous, pulsed feeding was used, rather than continuous. Constant and pulsed feeding have been directly compared for *R. toruloides* Y4 in complex medium, for which continuous feeding was shown to give slightly better lipid accumulation (62 g L^−1^ at 0.57 g L^−1^ h^−1^) than pulsed feeding [16]. Pulsed glucose addition was also used with *R. minuta*[7]. However, although ~65 g glucose L^−1^ were provided, biomass did not accumulate above 17 g L^−1^. A repeated batch process has been shown to be effective in sustaining lipid production by *R. glutinis* at low biomass concentration from palm oil effluent [13].

In this study *R. toruloides* was grown to high cell density in batch or fed-batch cultures in chemically defined medium with glucose, xylose or arabinose alone or in combination providing the carbon source. Continuous and discontinuous feed supply with or without nitrogen was considered for the fed-batch cultures, using C/Ns which allowed good lipid production rates in batch cultures. The results demonstrated that both batch and fed-batch cultures could be similarly efficient for lipid production by *R. toruloides* and that the presence of arabinose may have a strong negative effect on lipid production.

## Results

**Figure 1:**
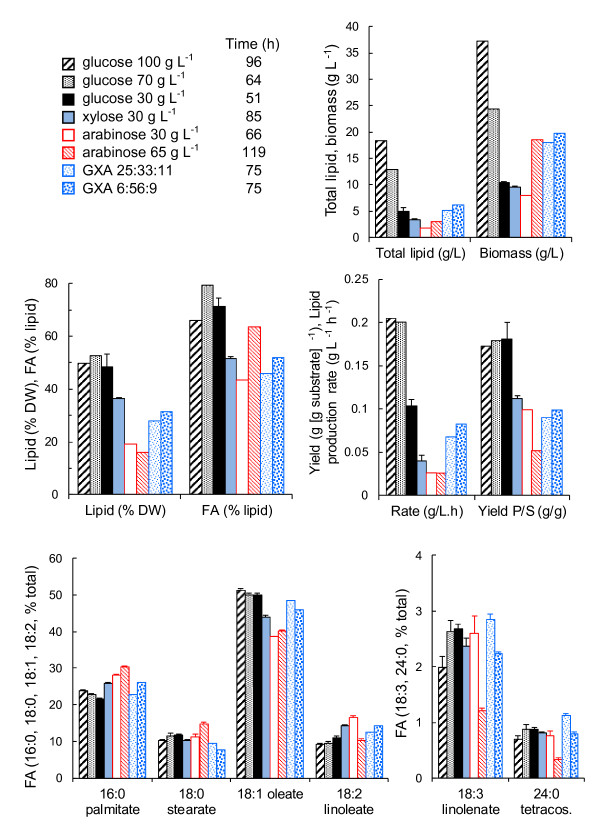
**Lipid production from glucose, xylose and/or arabinose by *R. toruloides* in batch culture.** Lipid (% DW and total produced), fatty acids (FA, % total lipid), biomass, rate of lipid production and yield of lipid on substrate at the time (indicated in the legend) of maximum total lipid production for *R. toruloides* growing in defined medium with glucose (black), xylose (light blue) or arabinose (red/white), or mixtures of glucose, xylose and arabinose (GXA) as carbon sources. Carbon was provided at 30 g L^−1^ (solid), 65–70 g L^−1^ (glucose, arabinose and GXA) or 100 g L^−1^ (glucose) at C/N 65 with (NH_4_)_2_SO_4_ as nitrogen source (pH 4, 30°C). The ratio of glucose, xylose and arabinose is given in the legend. The percentage of various fatty acids in the total fatty acids is also shown. Error bars represent ± SEM. Where error bars are not visible for fatty acids as % total SEM was too small to be seen at the scale shown.

Lipid production was compared at the time of maximum total (g L^−1^) lipid production. For batch cultures these times are reported in Table 1 and Figure 1. Typically maximum total lipid in batch culture was observed at the time when all carbohydrate had been consumed (start of stationary phase). However, in arabinose containing cultures, lipid content decreased before total consumption of the arabinose and thus while cells were still growing.

**Table 1:** Comparison of lipid production by *R. toruloides* CBS14 at different C/N in batch culture

**Carbon**	**C/N**	**Lipid production rate (g L**^**−1**^** h**^**−1**^**)**	**Yield lipid on carbohydrate(g g**^**−1**^**)**	**Lipid content (% DW)**	**Biomass (g L**^**−1**^**)**	**Time at which maximum lipid produced (h)**
Xylose	40	0.03	0.08	24	11	73
	65	0.04	0.11	36	10	85
	100	0.04	0.16	45	8	113
Glucose	40	0.08	0.10	23	9	26
	65	0.10	0.18	50	10	49
	100	0.08	0.20	56	9	66

Lipid production rate, yield and percent dry weight and biomass concentration were determined at the time of maximum total lipid produced. Carbon was provided as 30 g L^−1^ glucose or xylose and (NH_4_)_2_SO_4_ was provided as nitrogen. Cultures were grown in defined medium at pH 4.0, 30°C, 900 rpm, 1.2 vvm aeration.

### Characterisation of lipid production with glucose, xylose or arabinose as carbon sources at low substrate concentrations

The lipid content in the biomass increased with increase in the C/N from 40 to 100 with both glucose and xylose as carbon source (30 g L^−1^), but lipid content at high C/N was greater with glucose than with xylose (Table 1). The yield of lipid on substrate also increased with increasing C/N and was higher on glucose than on xylose (Table 1). Approximately 10 g biomass L^−1^ was produced from 30 g substrate L^−1^, but biomass production was slightly (p < 0.05) reduced at C/N 100 compared to C/N 65 (Table 1). The highest rate of lipid production was observed at C/N 65 on glucose, but was similar at C/N 65 and 100 on xylose (Table 1). Since lipid production rates were similar or higher at C/N 65 than at C/N 100 and culture times were shorter, further experiments were carried out with C/N 65. Corn steep solids could be partially or completely substituted for ammonium as the nitrogen source with no effect on lipid production, lipid production rate or yield of lipid on substrate (data not shown).

Maximum lipid and fatty acid production from glucose, xylose and arabinose were compared at C/N 65 with 30 g carbohydrate L^−1^ (Figure 1, solid black, blue and white bars). *R. toruloides* CBS14 accumulated a higher proportion of lipid in the biomass when grown on glucose (48%) than on either xylose (36%) or arabinose (19%), and a higher proportion of the lipid was made up of fatty acids. Lipid production rate and yield were also higher on glucose than on xylose or arabinose (Figure 1). The fatty acids produced on arabinose contained more 16:0 (palmitic, 28 ± 0.2%) and 18:2 (linoleic, 16.5 ± 0.5%), and less 18:1 (oleic, 38.6 ± 0.1%) fatty acids, than fatty acids produced on glucose (21.6 ± 0.2%, 11.0 ± 0,4% and 50.0 ± 0.4%, respectively) or xylose (Figure 1).

The specific growth rate was much lower on arabinose (μ = 0.14 h^−1^) and xylose (0.14 h^−1^) than on glucose (0.32 h^−1^) and less biomass was produced (Figure 1).

### High substrate concentration in batch culture

**Figure 2:**
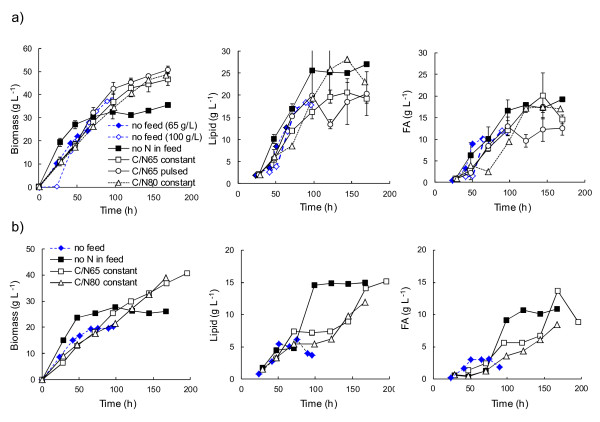
**Biomass, total lipid and total fatty acid production of *R. toruloides* in fed-batch cultures.** Cells were grown in defined medium with a) glucose and b) glucose, xylose and arabinose (6:55:8) provided as carbon sources in fed-batch cultures with (NH_4_)_2_SO_4_ as nitrogen source (pH 4, 30°C). The C/N during the batch phase for cultures which received no N in the feed was 40, whereas other cultures received the same C/N during batch and feeding phases (open symbols, C/N = 65 or 80, as indicated). Data for batch cultures (no feed) with glucose (70 or 100 g L^−1^, C/N 65) or glucose, xylose and arabinose (6:55:8, C/N 65) are also shown. Where shown, error bars represent ± SEM for duplicate cultivations. Error bars for biomass measurements include replicate measurements and are sometimes less than the size of the symbol.

Total biomass and lipid production increased in proportion with the concentration of glucose provided at C/N 65 (Figure 1, black bars). The proportion of lipid in the biomass and the yield of lipid from glucose remained approximately constant at 51% DW and 0.18 g lipid [g glucose consumed]^−1^, respectively (Figure 1). The lipid production rate was higher with 70 or 100 g glucose L^−1^ (0.20 and 0.21 g L^−1^ h^−1^, respectively) compared to 30 g glucose L^−1^ (0.10 g L^−1^ h^−1^). When cultures were provided 100 g glucose L^−1^ the lag phase was increased approximately 24 h compared to cultures with 30 or 70 g glucose L^−1^ (Figure 2), although the specific growth rate was not affected. We observed that a high rate of lipid synthesis (0.5 g L^−1^ h^−1^) was maintained between 50 and 75 h in cultures provided 100 g glucose L^−1^, and that the overall rate of lipid synthesis in these cultures was 0.33 g L^−1^ h^−1^ when the lag phase was excluded, suggesting that higher production rates could be achieved with 100 g glucose L^−1^ than with 70 g L^−1^ if osmotically adapted cells were used. None-the-less, to avoid long lag phases, ~70 g substrate L^−1^ was used in the batch phase of fed-batch cultures. Substrate concentration had little effect on the fatty acid profile (Figure 1).

**Figure 3:**
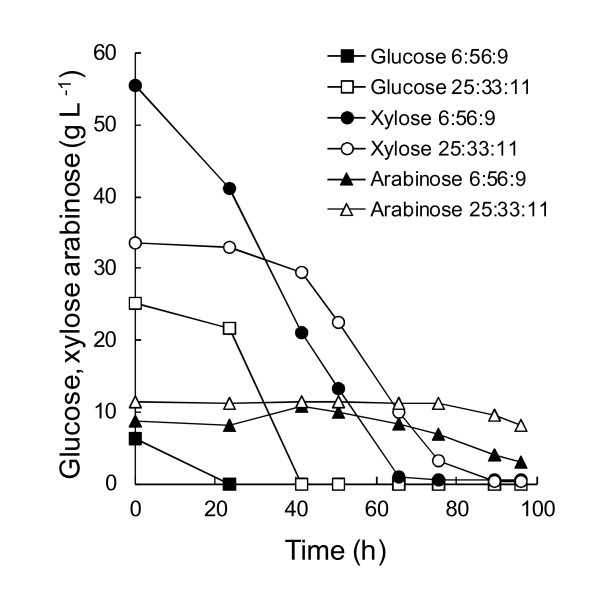
**Carbohydrate consumption by *R. toruloides* in batch culture with substrate mixtures.** Residual carbohydrates in cultures of *R. toruloides* grown in batch culture in defined medium with glucose, xylose and arabinose provided as carbon sources in the ratio of 25:33:11 (open symbols, 25:33:11) or 6:56:9 (solid symbols, 6:56:9) to a total of ~70 g L^−1^ at C/N 65 with (NH_4_)_2_SO_4_ as nitrogen source (pH 4, 30°C).

*R. toruloides* was also grown in mixtures of glucose, xylose and arabinose to provide a total of ~70 g carbohydrate L^−1^. One mixture contained 25 g glucose L^−1^, 33 g xylose L^−1^ and 11 g arabinose L^−1^, similar to the ratio of sugars obtained from acid hydrolysed dried distillers grain solids (M.G. Wiebe, unpublished observation) with no separation of C5 and C6 carbohydrates. The second mixture contained 6 g glucose L^−1^, 56 g xylose L^−1^ and 9 g arabinose L^−1^, similar to the ratio of sugars expected in a C5-enriched hydrolysate. The carbohydrates were consumed consecutively, with glucose being consumed first, then xylose, and finally arabinose, which was not completely consumed (Figure 3). Biomass production was similar to that on arabinose (65 g L^−1^) alone (Figure 1).

*R. toruloides* produced less lipid (28–31% DW, 5–6 g L^−1^), at a lower rate (0.07-0.08 g L^−1^ h^−1^) and yield (0.09-0.10 g [g substrate consumed]^−1^), when grown on mixtures of glucose, xylose and arabinose, compared to growth on glucose (52% DW, 13 g L^−1^) as sole carbon source, although more lipid was produced than on arabinose (16% DW, 3 g L^−1^, Figure 1). Lipid content (% DW) and yield on carbohydrate were similar to that of cells grown on 30 g xylose L^−1^ (Figure 1). Cultures receiving a high proportion of glucose in the mixture did not produce more lipid than those with the low proportion of glucose. However, cells provided a higher proportion of glucose in the carbohydrate mixture had a profile of fatty acids which was more similar to those observed in glucose cultures than cells which received a high proportion of xylose and only low glucose in the mixture (Figure 1).

### Fed-batch strategies for lipid production with *R. toruloides*

Four strategies were tested for lipid production in fed-batch cultures with *R. toruloides* CBS14 (Figure 2), using either glucose alone or a mixture of glucose, xylose and arabinose (6:55:8). In one strategy, nitrogen was provided in the batch phase at C/N 40, comparable to the lowest C/N tested in batch cultures, but none in the feed, resulting in a gradual reduction in the C/N to ~99. For the other three strategies, nitrogen was provided at C/N 65 in the batch phase, as in high cell density batch cultures, or C/N 80, and also at the same C/N in the feed. Providing C/N 40 in the batch phase enabled more rapid accumulation of biomass than with C/N 65 or 80. Lipid and fatty acid accumulation was also high using this strategy, particularly with the mixture of substrates, but biomass (35 g L^−1^ on glucose, 27 g L^−1^ on mixed substrates) and lipid (25 g L^−1^ on glucose, 15 g L^−1^ on mixed substrates) production stopped after 70–100 h and not all substrate was consumed (Figure 2). The biomass contained up to 75% lipid on glucose and 58% lipid on mixed substrates (Table 2).

**Table 2:** Comparison of lipid production by *R. toruloides* CBS14 in batch and fed-batch cultivation

**Carbon**	**C/N in batch**	**C/N in feed**	**Lipid production rate (g L**^**−1**^** h**^**−1**^**)**	**Yield lipid on carbohydrate (g g**^**−1**^**)**	**Lipid content (% DW)**
Glucose (65 g L^−1^)	65	no feed	0.21	0.18	50
	65	C/N 65	0.16	0.13	50
	65	C/N 65 (pulsed)	0.18	0.15	47
	80	C/N 80	0.20	0.15	61
	40	no N in feed	0.21	0.22	75
Glucose, xylose, arabinose (6:55:8)	65	no feed	0.08	0.10	31
	65	C/N 65	0.08	0.08	38
	80	C/N 80	0.07	0.06	31
	40	no N in feed	0.15	0.07	58

Lipid production rate, yield and percent dry weight were determined at the time of maximum total lipid produced (g L^−1^, see Figure 2). Carbon was provided as glucose or a mixture of glucose, xylose and arabinose (6:55:8) and (NH_4_)_2_SO_4_ was provided as nitrogen. Cultures were grown in defined medium at pH 4.0, 30°C, 900 rpm, 1.2 vvm aeration.

Since high rates of lipid production were observed in batch cultures with C/N 65, fed-batch cultures with constant provision of C/N 65 were also tested. Fed-batch cultures with C/N 80 were tested to determine whether lipid content in the biomass would be increased, as predicted from batch cultures. Biomass production was sustained throughout the cultivation when nitrogen was provided in the feed at C/N 65 or 80 (Figure 2) and 47–51 g biomass L^−1^ were produced from glucose, 39 g biomass L^−1^ from mixed substrates, regardless of the C/N ratio or whether the feed was provided at a constant rate or in pulses. The lipid content of the biomass was lower than in the cultures which received no N in the feed (Table 2), but total lipid (21 g L^−1^ and 15 g L^−1^ from glucose and mixed substrates, respectively) and fatty acid (20 g L^−1^ and 14 g L^−1^, respectively) production was similar to that in the cultures with no N in the feed (Figure 2). Substrates were more completely utilised in cultures which received N in the feed than in those which did not. Increasing the C/N from 65 to 80 resulted in higher concentrations of residual substrate(s) and lipid content in the biomass was only slightly increased with glucose as substrate, but not with glucose, xylose and arabinose (Table 2). Lipid production was similar, but fatty acid production was slightly less efficient when the substrate was provided in pulses, rather than continually (Figure 2, Table 2).

The yield of lipid on carbohydrate was reduced in all fed-batch cultures, compared to batch cultures, with the exception of glucose fed cultures which received no N in the feed (Table 2). Lipid production rates were generally similar in fed-batch cultures to those observed in comparable batch cultures (Table 2). However, on mixed substrates provided C/N 40 during the batch phase and no N in the feed the lipid production rate was 0.15 g L^−1^ h^−1^, which was almost as high as on glucose alone (Table 2).

**Figure 4:**
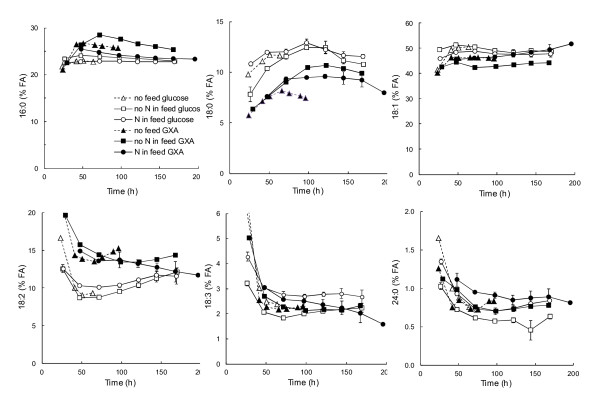
**Fatty acid composition of *R. toruloides* in fed-batch cultures.** Cells were grown in defined medium with glucose (open symbols) or a mixture of glucose, xylose and arabinose (6:55:8, solid symbols) provided as carbon sources with (NH_4_)_2_SO_4_ as nitrogen source (pH 4, 30°C). The C/N during the batch phase for cultures which received no N in the feed (squares) was 40, whereas cultures with N in the feed (circles) received C/N 65 or 80 during both batch and feeding phases. Data for all cultures receiving N in the feed (C/N 65 or 80, constant or pulsed feed) have been combined and error bars represent ± SEM for 2–5 cultivations. Data for batch cultures (no feed, triangles) with glucose (70 g l^−1^, C/N 65) or glucose, xylose and arabinose (6:55:8, C/N 65) are also shown.

The feeding strategy (C/N 65 or 80, pulsed or constant) did not affect the proportions of fatty acids extracted from the biomass (Figure 4). Provision of xylose and arabinose in the feed in addition to glucose generally reduced the proportion of 18:0 fatty acids and increased the proportion of 16:0 (when no nitrogen was added to the feed) and 18:2 fatty acids, compared to cultures receiving glucose alone (Figure 4). Fatty acid profiles were similar to those of batch cultures receiving the same carbon source(s) (Figure 4).

## Discussion

*R. toruloides* is a known lipogenic yeast and the results presented here confirm its ability to accumulate over 75% of its biomass as lipid, with 62% as fatty acids. However, lipid accumulation was strongly affected by the nature of the carbon source provided. Greater lipid accumulation occurred on glucose or xylose than on arabinose (Figure 1), as has also been observed for *Trichosporon cutaneum* (reported in [1]). On mixtures of carbohydrates, lipid accumulation was lower than on glucose, but only slightly lower than observed on xylose (Figure 1), reflecting the sequential utilisation of the sugars, with glucose being consumed while more nitrogen was available and xylose and arabinose being consumed during the period of nitrogen restricted growth. Further, arabinose consumption was incomplete, and thus the effective C/N was less than intended, contributing to the reduced lipid accumulation. Interestingly, lipid production was similar regardless of whether the ratio of glucose to xylose and arabinose was high (25:35:11) or low (6:56:9). The low lipid production on mixtures of carbohydrates should be taken into account when assessing the possibilities of producing lipids directly from plant biomass hydrolysates. Low lipid content in *R. glutinis* (6–29% lipid per g biomass, [5]) and *R. toruloides* (25% lipid per g biomass, [14]) has indeed been observed when lipids have been produced directly from plant hydrolysates.

It is also important to note that the fatty acid profile was not the same on mixtures of glucose, xylose and arabinose as on glucose alone. Thus in plant biomass hydrolysates one should expect a lower proportion of stearic acid (18:0), and more linoleic (18:2) and possibly more palmitic (16:0) fatty acids than in pure glucose. Some caution should be taken when extrapolating results from glucose grown yeast to what can be expected from plant biomass hydrolysate. Although arabinose was not generally completely metabolised its presence in the medium appeared to have a strong influence on the fatty acid profile.

*R. toruloides* CBS14 (lipid production rate ~0.2 g L^−1^ h^−1^ from glucose) was not as productive as *R. toruloides* Y4 (lipid production rate ~0.5 g L^−1^ h^−1^ from glucose; [4,16]). This may reflect difference between the strains, the presence of yeast extract (and peptone) in the Y4 processes, and/or a lower C/N during the batch phase for the Y4 processes [4,16]. Although CBS14 has been studied extensively as a model of lipid production in yeast, it is not necessarily the best strain for production. It is none-the-less worth noting that during the latter stages of batch culture with 70 or 100 g glucose L^−1^ it was producing lipid at 0.5 g L^−1^ h^−1^, indicating that it has more potential than has so far been achieved in batch or fed-batch cultivations.

High lipid accumulation occurred on chemically defined medium and did not require addition of expensive complex ingredients such as yeast extract, even in high cell density batch or fed-batch cultures. If a complex nitrogen source is desired, CSS were found to be suitable for good lipid production. Although most studies of lipid production from waste streams have included yeast extract or peptone in the medium, Xue et al. [12] demonstrated that no nitrogen or other supplements were needed to produce lipid in *R. glutinis* on monosodium glutamate wastewater.

The inhibitory effect of high (100 g L^−1^) glucose concentrations in batch culture has also been observed by Li et al. [4] for *R. toruloides* Y4, but only for concentrations above 150 g L^−1^. This inhibition could be avoided by using a repeated or sequential batch process in which a large proportion (e.g. 50–90%) of the culture would be removed and replaced with fresh medium at the time when lipid and biomass content would be maximal, as illustrated with *R. glutinis* on palm oil mill effluent [13]. Alternatively, inhibition by high substrate concentrations could be avoided by utilising fed-batch and repeated fed-batch cultivation, as shown here and for *R. toruloides* Y4 [4,16] and *C. curvatus*[15].

As reported by Zhao et al. [16] for *R. toruloides* Y4, we found that continuous feeding was preferable to discontinuous, intermittent feeding for high fatty acid accumulation in CBS14 (Figure 2). We also observed that the best rates and yields of lipid production were achieved using glucose feed which lacked nitrogen (Table 2). However, in contrast to Li et al. [4], Zhao et al. [16], and Hassan et al. [15], biomass production was not sustained under these conditions (Figure 2), substrate was not completely consumed and the rates and yields would not be expected to be sustainable in a repeated fed-batch process. This probably reflects the absence of yeast extract or other complex ingredients in our process, along with the use of a higher initial C/N during the batch phase than has been used previously. If reduction of the organic carbon content of the feed is also important, providing nitrogen in the feed could be beneficial, even though there could be some loss in yield. This is even more apparent if the fatty acid content is taken into account, rather than just the total lipid, since the rate of fatty acid production was similar with or without nitrogen in the feed (Figure 2a). However, it is likely that either process would benefit from a lower C/N during the batch phase, to enable more rapid biomass accumulation. It is also likely that feed derived from plant biomass hydrolysates will always contain some metabolisable nitrogen. Further, a repeated batch process with e.g. 100 g glucose L^−1^, C/N 65, would be more efficient in converting glucose to lipid if a rate of 0.33 g lipid L^−1^ h^−1^ would be sustained without the lag phase.

In contrast, fed-batch cultivation with no nitrogen in the feed was clearly the best option for producing lipids from mixed carbohydrate substrates including glucose, xylose and arabinose (Table 2, Figure 2b). As with glucose fed-batch cultures, one can predict that a lower C/N in the batch phase, possibly combined with a high C/N feed, would be necessary to achieve more complete utilisation of the substrate and to create a process which would be sustainable as a repeated fed-batch process.

## Conclusions

Lipid production from C5 carbohydrates was less efficient in *R. toruloides* CBS14 than from glucose, with production from xylose being better than from arabinose. The presence of arabinose in media containing mixtures of glucose, xylose and arabinose resulted in less lipid accumulation than in medium with either glucose or xylose alone. Thus plant biomass hydrolysates with low arabinose content would be desirable for lipid production using *R. toruloides* CBS14.

High biomass and lipid production were achieved in both batch and fed-batch cultures with glucose as carbon source, and a repeated batch process would be expected to be as efficient as a fed-batch or repeated fed-batch process. However, for lipid production from mixtures of carbohydrates, fed-batch cultivation was preferable. In fed-batch processes, provision of extra nitrogen during the batch phase was useful to provide rapid biomass production, with lipid accumulating during the feeding phase. Provision of nitrogen in the feed enabled more complete utilisation of the carbohydrate(s), but with a lower yield of lipid on carbohydrate, than in its absence. Complete substrate utilisation would be important in lipid production from waste streams, to ensure sufficient reduction in the carbon content of the stream. The feeding strategy did not affect the relative proportion of different fatty acids in the lipid, but the presence of C5 carbohydrates did.

## Methods

### Strain

*Rhodosporidium toruloides* CBS14 was obtained from CBS and maintained as cells in 15% (v/v) glycerol with 0.9% (w/v) NaCl at −80°C.

### Medium

Cells were maintained on YPD (yeast extract 10 g L^−1^, peptone 20 g L^−1^, glucose 20 g L^−1^) solidified with 15 g agar L^−1^.

The defined medium for lipid production contained 1.2 g KH_2_PO_4_ L^−1^, 0.3 g Na_2_HPO_4_ L^−1^, 1.5 g MgSO_4_ L^−1^, 0.1 g CaCl_2_·6H_2_O L^−1^, 5.26 mg citric acid·H_2_O L^−1^, 5.26 mg ZnSO_4_·7H_2_O L^−1^, 0.1 mg MnSO_4_·4H_2_O L^−1^, 0.5 mg CoCl_2_·6H_2_O L^−1^, 0.26 mg CuSO_4_·5H_2_O L^−1^, 0.1 mg Na_2_MoO_4_·2H_2_O L^−1^, 1.4 mg FeSO_4_·7H_2_O L^−1^, 0.1 mg H_3_BO_4_ L^−1^, 125 mg meso-inositol L^−1^, 5 mg nicotinic acid L^−1^, 6.2 mg calcium pantothenate L^−1^, 5 mg thiamine HCl L^−1^, 6.2 mg pyridoxine HCl L^−1^, and 0.125 mg biotin L^−1^. Nitrogen was provided as (NH_4_)_2_SO_4_ or CSS and carbon as glucose, xylose or arabinose. The carbon-nitrogen ratio (C/N) determined the amount of nitrogen which was added to the medium. Inoculum for batch and fed-batch cultures was grown in the lipid production medium with urea substituted for (NH_4_)_2_SO_4_ to provide buffering.

### Culture conditions

Cells were maintained on agar-solidified medium in 9 cm diam. Petri dishes. Inocula for batch and fed-batch cultures were grown in 250 mL flasks containing 50 mL medium and agitated at 200 rpm, 30°C.

Batch and fed-batch bioreactor cultures were carried out in Multifors bioreactors (max. working volume 500 mL, Infors HT, Switzerland) containing 200–500 mL medium. Cultures were maintained at 30°C, 900 rpm, with 1.2 volume gas (volume culture)^−1^ min^−1^ (vvm). Culture pH was kept constant at pH 4.0 by the addition of sterile 1 M KOH or 1 M H_3_PO_4_. Clerol FBA 3107 antifoam (Cognis France, Ponthierry Paris; 0.01% v/v for batch cultures) or polypropylene glycol (mixed molecular weight [17], 0.1% v/v for fed-batch cultures) was added to control foam production. Gas concentration (CO_2_, O_2_, N_2_ and Ar) was analysed continuously in an Omnistar quadrupole mass spectrometer (Balzers AG, Liechtenstein), calibrated with 3% CO_2_ in Ar.

In fed-batch cultures, the feed medium was provided as a concentrated solution of carbohydrate, with or without (NH_4_)_2_SO_4_, at C/N 65 or 80 at a constant rate of 2 mL h^−1^ or intermittently as a 48 mL pulse at approximately 24 h intervals using the Multifors built in feed pump. Feed medium also contained trace elements and vitamins (concentrated four-fold compared to medium for batch cultures) and Clerol FBA 3107 antifoam (1 mL L^−1^).

### Measurement of biomass

Biomass was measured as optical density at 600 nm (OD_600_) or as dry weight (DW). For dry weight, samples were collected in 2 mL pre-dried, pre-weighed micro-centrifuge tubes, washed twice with equal volume distilled water and dried at 100°C.

### HPLC analyses

The concentration of glucose, xylose, arabinose, and glycerol were determined by HPLC using a Fast Acid Analysis Column (100 mm × 7.8 mm, BioRad Laboratories, Hercules, CA) linked to an Aminex HPX-87H organic acid analysis column (300 mm × 7.8 mm, BioRad Laboratories) with 2.5 mM H_2_SO_4_ as eluent and a flow rate of 0.5 mL min^−1^. The column was maintained at 55°C. Peaks were detected using a Waters 410 differential refractometer and a Waters 2,487 dual wavelength UV (210 nm) detector and the results processed using Waters Empower software.

### Lipid extraction and analyses

Lipids were extracted using a chloroform-methanol method [18], modified for small-scale extractions. Biomass (20–50 mg dry biomass) was collected by centrifugation of a suitable volume of culture broth, washed with an equal volume of de-ionised water, and stored at −40 or -80°C in Eppendorf Safe-Lock micro-centrifuge tubes. Biomass was extracted in 0.5 mL methanol containing 0.1% (w/v) butylated hydroxytoluene, using a chilled Teflon adapter in a Mixer Mill MM 301 (Retsch GmbH, Germany) with two 5 mm zirconium oxide and three 3 mm yttrium stabilised zirconium oxide balls (5 min at 20 Hz) to disrupt the cells after incubation on ice for 10 min. Homogenisation was repeated after cooling the extracts and adapters for at least 5 min. Cold chloroform (1 ml) was added, along with triheptadecanoin (1.6 μg μL^−1^) and heptadecanoic acid (1.6 μg μL^−1^) as internal standards. The sample was transferred to a clean tube with 300 μL acetic acid and vortexed for 5–10 min at high speed. The lower phase was collected after centrifugation and the upper phase washed with 2 parts chloroform. The combined organic phases from the sample were dried under nitrogen and lipid was determined gravimetrically. Lipid was re-dissolved in 0.5 mL cold chloroform: methanol containing 0.1% w/v butylated hydroxytoluene (2:1) for storage at −20°C.

For GC analysis of the fatty acids, the chloroform: methanol was evaporated under nitrogen from an aliquot of sample, which was re-dissolved in 0.7 mL petroleum ether. After vortexing, 250 μL sodium methoxide in dry methanol (0.5 M) was added and the sample heated at 45°C for 5 min after mixing. The sample was mixed with NaHSO_4_ (0.5 mL 15% w/w) and petroleum ether (0.2 mL) and the petroleum ether layer collected after centrifugation. Petroleum ether was removed by evaporation and the sample dissolved in 0.1 mL hexane, which was analysed on a Hewlett Packard HP 5890 Series II GC. Samples in hexane (2 μL) were analysed on an Agilent capillary column (25 m × 0.20 mm, 0.33 μm coating thickness). The oven temperature was maintained for 1 min at 70°C, then increased linearly to 240°C at a rate of 7°C min^−1^ with helium as the carrier gas.

## Competing interests

The authors declare that they have no competing interests.

## Authors’ contributions

Experimental work and analyses were carried out by MGW (bioreactor cultivations) and KK (lipid analyses). MGW wrote the manuscript, which was reviewed by KK and LR. MGW, KK, LR and MP all contributed to the experimental design and read and approved the final manuscript.
